# Hydroalcoholic Extracts from *Pleurotus ostreatus* Spent Substrate with Nematocidal Activity against *Nacobbus aberrans* Phytonematode and the Non-Target Species *Panagrellus redivivus*

**DOI:** 10.3390/plants13131777

**Published:** 2024-06-27

**Authors:** Julio Cruz-Arévalo, Víctor M. Hernández-Velázquez, Alexandre Toshirrico Cardoso-Taketa, Manases González-Cortazar, José E. Sánchez-Vázquez, Guadalupe Peña-Chora, Edgar Villar-Luna, Liliana Aguilar-Marcelino

**Affiliations:** 1 Centro de Investigación en Biotecnología, Universidad Autónoma del Estado de Morelos, Cuernavaca 62209, Morelos, Mexico; julio.cruz@uaem.edu.mx (J.C.-A.); ataketa@uaem.mx (A.T.C.-T.); 2 Centro de Investigaciones Biomédicas del Sur, Instituto Mexicano del Seguro Social, Argentina No. 1 Centro, Xochitepec 62790, Morelos, Mexico; gmanases@hotmail.com; 3 El Colegio de la Frontera Sur, Carretera al Antiguo Aeropuerto km 2.5, Tapachula 30700, Chiapas, Mexico; esanchez@ecosur.mx; 4 Centro de Investigaciones Biológicas, Universidad Autónoma del Estado de Morelos, Cuernavaca 62209, Morelos, Mexico; penacg@uaem.mx; 5 Instituto Politécnico Nacional, Centro Interdisciplinario de Investigación para el Desarrollo Integral Regional, Unidad Michoacán, Jiquilpan 59510, Michoacán, Mexico; evillarl@ipn.mx; 6 CENID-Salud Animal e Inocuidad, Instituto Nacional de Investigaciones Forestales Agrícolas y Pecuarias, Jiutepec 62550, Morelos, Mexico

**Keywords:** bioactivity, pest control, agro-industrial residues, myco-chemical

## Abstract

*Pleurotus ostreatus*, an edible mushroom widely consumed worldwide, generates a by-product known as spent mushroom substrate (SMS). This material has demonstrated biological activity against agricultural crop pathogens. In this study, we evaluated the nematocidal effectiveness of hydroalcoholic extracts (T5, T2, AT5, and AT2) derived from SMS of *P. ostreatus* against (J_2_) of the phytonematode *Nacobbus aberrans* and assessed their potential toxicity towards the non-target nematode *Panagrellus redivivus*. Among these extracts, AT5 exhibited the highest efficacy against *N. aberrans* and was the least toxic against *P. redivivus*. Liquid–liquid partitioning yielded the AQU fraction, which showed significant nematocidal activity against J_2_ (75.69% ± 8.99 mortality), comparable to chitosan. The GC-MS analysis revealed the presence of several compounds, including palmitic acid, linoleic acid, and 2,4-Di-tert-butylphenol. These findings are consistent with studies confirming the antagonistic effectiveness of these compounds against phytonematodes. Additionally, all extracts exhibited toxicity against *P. redivivus*, with T2 being the most toxic. Our findings demonstrate that while the AT5 extract displays antagonistic effectiveness against both *N. aberrans* and *P. redivivus*, it was the least toxic among the extracts tested. Thus, SMS of *P. ostreatus* holds potential as a source of nematocidal compounds, which could offer significant benefits for agricultural pest control.

## 1. Introduction

Edible mushrooms (EM) are currently cultivated in more than 100 countries, raising a growth rate of 6–7% per year [1]. *Pleurotus* spp. are among the most produced; they can be cultivated on different agro-industrial residues [2,3]. Each kilogram of fresh edible mushroom produces 5–6 kg of spent mushroom substrate (SMS); however, this material still has the following benefits: as fuel, compost, organic amendment, animal feed, and mulch material, among others [4,5]. The residuals of SMS consist of mycelial material, low commercial-quality mushrooms or primordia, and depleted substrate [6].

Several studies have demonstrated that SMS contains active compounds, including phenolic compounds, flavonoids, alkaloids, organic acids, anthocyanins, polyketides, terpenoids, and steroids [7]. The biological activity of spent substrate from different EM species has been documented on agricultural crop pathogens such as bacteria, insects, fungi, and nematodes [8,9,10,11]. Regarding the latter, nematocidal activity (NAT) against gastrointestinal, plant-parasitic, and free-living nematodes (FLNs) have been reported. For example, Tanimola et al. [11] reported 100% mortality and inhibition of eggs (IEH) of *Meloidogyne incognita* and *M. javanica* (J_2_) exposed in vitro to aqueous extracts of spent substrate of *Pleurotus* spp. Likewise, Hahn et al. [12] observed the same effect on *M. javanica* confronted with aqueous extracts of *P. eryngii* spent substrate. In addition, in vitro NAT of the SMS of *Pleurotus* spp. has been reported against *Haemonchus contortus* L_3_, a hematophagous gastrointestinal nematode [13,14]. 

Other studies have revealed the NAT of the SMS crude extract of *Hypsizygus marmoreus* against infective larvae (L_3_) bovine and the FLN *Panagrellus redivivus*. In this study, the NAT was mainly attributed to proteases [15]. In another study conducted by Genier et al. [16], the destruction or digestion of *P. redivivus* larvae exposed in vitro to *P. ostreatus* proteases was shown. Kwok et al. [17] isolated from the SMS of *P. ostreatus* the trans-2-decenedioic acid, a nematocidal molecule against *P. redivivus*. Heydari et al. [18] also detected toxins in the mycelium of five *Pleurotus* species tested in vitro against *M. javanica* J_2_. Likewise, several studies suggest that fatty acids are related to the NAT of *Pleurotus* spp. against nematodes with different types of feeding strategies [19,20,21,22].

Despite the previously mentioned evidence, no studies are reporting the NAT of SMS of EM against *Nacobbus aberrans* (false root-knot nematode), one of the ten most important phyto-nematodes worldwide [23]. *Nacobbus aberrans* is polyphagous and can infect up to 84 plant species, including agricultural crops and ornamental plants [24,25]. It is associated with crops and plants native to temperate and subtropical regions of North and South America [26]. In addition, *N. aberrans* is a pest registered as of quarantine importance in the United States and Europe. During the infection process, phyto-nematodes can injure crops by diverting nutrients, disturbing water transport, and triggering potential attacks from other pathogens like fungi, bacteria, and viruses [27].

Natural pesticides may have a negative impact on non-target organisms (NTOs), and they are environmentally safe in most cases; however, it is not always that way; for example, nicotine is from natural origin, but it is far more toxic than most modern synthetic pesticides [28]. For this reason, it is very important to test the ecotoxicological impact of natural and synthetic pesticides. The FLNs have an essential role in the nutrient cycle of soils; due to their eating habits and their excretions, they can contribute with 8–19% of the soluble N of the soil. In addition, the abundance of FLN has been related to concentrations of other nutrients in the soil [29]. FLNs are good food sources for other organisms and facilitate the transport and dispersal of bacteria in the soil [30]. They also contribute to the decomposition of dead matter, allowing plants to have better accessibility to nutrients [31]. The nematode *P. redivivus* is a test organism applicable to the biomonitoring of water and sediments [32]; however, it can also be used as an indicator of the toxicity of natural products since some substances of natural origin can be toxic even to humans [33].

In summary, the observed nematocidal properties of *Pleurotus* spp. and its spent substrate suggest that this material may have an antagonistic effect against the phyto-parasite *N. aberrans*. Therefore, in the present study, we evaluated the nematocidal effectiveness of hydroalcoholic extracts of spent substrate from *P. ostreatus* against J_2_ of *N. aberrans,* while also identifying the compounds present in an active fraction using GC-MS. In addition, we tested the toxicity against *P. redivivus* to screen for some negative effects of the extracts.

## 2. Results

### 2.1. Effectiveness of Extracts against J_2_ of N. aberrans

**Figure 1:**
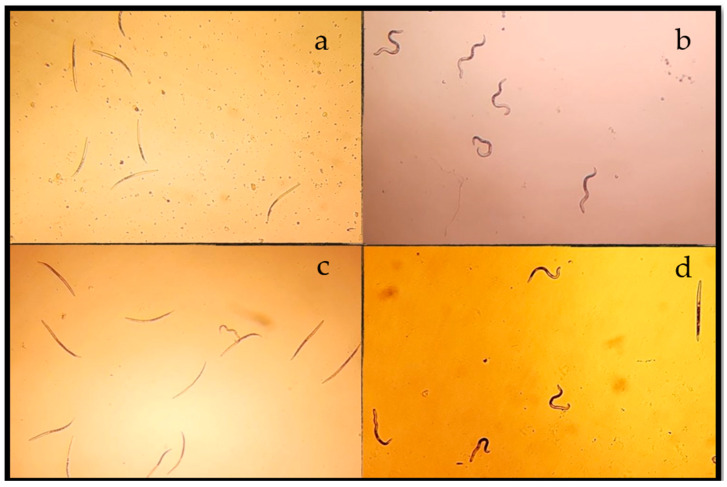
This description explains the visual distinction between living and dead J_2_ under a microscope (40× magnification) after treatment with NaOH 1N. Live nematodes appear wavy, while dead ones appear stretched. The specific treatments tested include (**a**) positive control (chitosan), (**b**) negative control (water), and two test treatments, where (**c**) AT5 (10 mg/mL) and (**d**) AT2 (20 mg/mL) show live and active nematodes.

Table 1 shows the effectiveness of extracts against *N. aberrans*; the LC_50_ values indicate that T2 and T5 treatments were the most effective. Likewise, the most effective extract containing fungal material was AT5. When comparing both colonized extracts, the LC_50_ of AT5 (2.73 mg/mL) was closest to that of the commercial chitosan nematicide (Figure S1). Therefore, AT5 was selected for further in vitro testing at the fraction level. On the other hand, NaOH 1N stimulated J_2_ and helped accurately to determine the mortality; the wavy appearance served as a reference to confirm living J_2_ (Figure 1).

### 2.2. Nematocidal Effectiveness of the Fractions against J_2_ of N. aberrans

Larval mortality of J_2_ exposed in vitro to three different fractions from AT5 was performed. Fractions were tested at a concentration of 1 mg/mL to screen for the most lethal. The best was the AQU fraction, which reached a mean mortality of 87.95% ± 5.26 on J_2_. The lowest treatment was the DM fraction; however, it was different compared to the negative control (Table 2).

### 2.3. Comparison of Nematocidal Activity and Hatching Inhibition between the AQU Fraction and Commercial Chitosan

**Figure 2:**
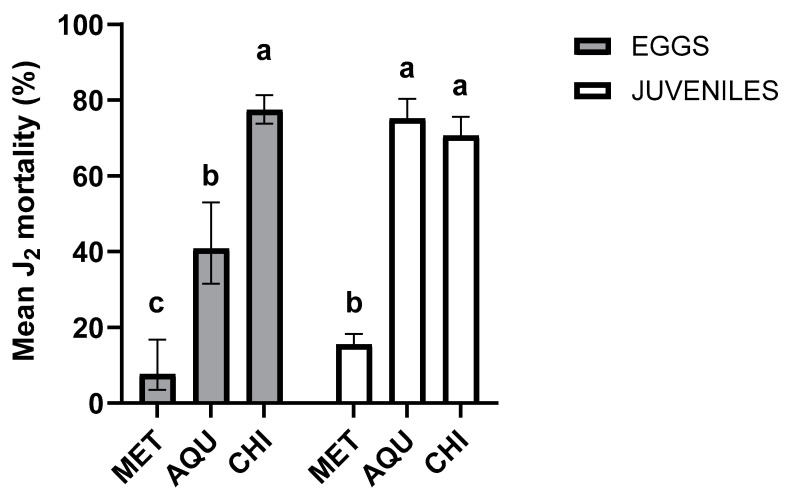
Effectiveness of AQU fraction (1 mg/mL) and Chitosan (CHI) compound (6 mg/mL) against J_2_ and eggs of *N. aberrans*. The scatter plots indicate the 95% confidence interval. Averages with different letters indicate significant differences (Tukey, *p* < 0.05). Eggs F_(2, 15)_ = 129.1, *p* > 0.0001; juveniles (F_(2, 42)_ = 296.8); *p* > 0.0001.

Figure 2 illustrates the nematocidal effect of the AQU fraction against eggs and J_2_ of *N. aberrans*. According to the Tukey test (*p* < 0.05), the AQU fraction’s effectiveness against J_2_ was comparable to that of CHI (6 mg/mL). However, when tested against eggs, its effectiveness was reduced by half compared to its larvicidal effectiveness. Nematocidal effectiveness is considerable because the concentration assessed was six times lower than chitosan.

### 2.4. Identification of Compounds Present in AQU Fraction

**Figure 3:**
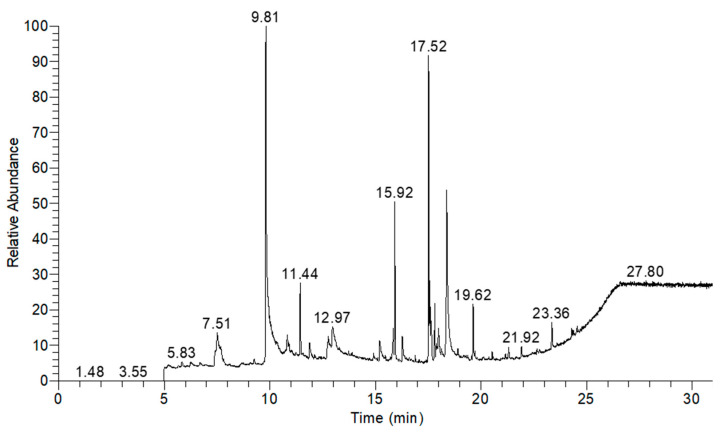
GC-MS chromatogram of AQU fraction derived from AT5 extract.

Table 3 shows the structures of the eight compounds detected in the AQU fraction through GC-MS analysis. The time retention ranged from 5–30 min (Figure 3), and the compounds belong to a range of chemical classes, including sugars, amino acid derivatives, phenols, fatty acid esters, and hopanoids, suggesting the diverse nature of the AQU fraction.

### 2.5. Toxicity of Extracts against P. redivivus

All the extracts reached the threshold of over 50% mortality (Table 4), so the four extracts are toxic against *P. redivivus.* The highest toxicity was observed in the T2 extract, reaching a LC_50_ of 1.37 (1.25–1.51 mg/mL), and in contrast, the least was AT5 with a LC_50_ of 8.56 (7.04–10.09 mg/mL). In fact, both colonized substrates are less toxic against *P. redivivus* than those not spawned with *P. ostreatus*. The same pattern was noted in the LC_90_ values as well. T2 reaches 90% of larval mortality at 25.12 (19.34–30.92 mg/mL), while AT5 needs 93.15 (49.06–137.24 mg/mL) to reach such mortality.

## 3. Discussion

Four crude hydroalcoholic extracts obtained from *P. ostreatus* spent substrate were evaluated for their impact on the non-target *P. redivivus* nematode and *N. aberrans* phyto-nematode. The results suggest a dose-dependent effect. The LC_50_ and LC_90_ values indicate that the most effective extracts are those that were not colonized by *P. ostreatus* (as indicated in Table 1 and Table 2). It is possible that the fermentation induced by the mushroom contributed to reducing the concentration or eliminating active compounds in the colonized substrates. According to Carrasco et al. [34], *P. ostreatus* was capable of assimilating and degrading caffeine when cultivated in spent coffee grounds. Furthermore, it seems that the proportion of coffee pulp does not have a significant influence on the level of toxicity against *N. aberrans*, as the most toxic extracts were those with equal distribution of raw material. Our results contrast with those obtained by Castañeda-Ramírez [35], where no nematocidal activity was observed against *H. contortus* L_3_ larvae confronted against extracts from the same raw materials tested at the present study. 

Lower concentrations were required to achieve a 50% mortality rate for *N. aberrans* J_2_ compared to *P. redivivus*. This means that *N. aberrans* is more susceptible to the tested extracts. This aligns with the findings suggested by Gomesa et al. [36]. They observed a difference between the nematocidal effects of proteases against *M. incognita* and *P. redivivus*, and they proposed that efficacy is probably due to the cuticle differences. Among the spawned extracts, AT5 was the better with an LC_50_ of 2.73 mg/mL. There are few in vitro studies reporting the NAT of SMS extracts against phyto-parasitic nematodes. Most of the natural products assessed against *N. aberrans* are plant-derived products. Additionally, many of these studies are focused on *Meloidogyne* spp., the most important phyto-nematode worldwide. Mortality rates like our findings were observed in studies conducted by Hahn et al. [12], who investigated the effect of *P. ostreatus* spent substrate against *M. incognita* J_2_, reporting mortality from 50–77%. Additionally, Sosa et al. [37] reported mortality rates of 90–100% for *N. aberrans* when applying essential oils at concentrations of 800–1000 µL L^−1^.

According to Colmenares-Cruz et al. [14], during the solid fermentation process, many metabolites are released to the SMS, and this could influence the NAT. In their mycelial stage, fungi can be attacked by mycophagous nematodes such as *Aphelenchus avenae*, *Aphelenchoides* spp., and *Ditylenchus myceliophagus* [38]. Therefore, the metabolites produced as a defense strategy could be capable of killing or deterring pest organisms. In addition to the nematocidal effect observed against *N. aberrans*, the advantage of using SMS is that it can provide benefits through the bioconversion of waste into edible mushrooms. 

As mentioned above, AT5 was subjected to liquid–liquid partition due to the observed higher mortality between both spawned extracts. The AQU fraction was the most effective, and it was different statistically compared to DM and EAC (Table 3). These fractions also had considerable NAT, suggesting that both have toxic compounds against *N. aberrans*. The AQU fraction was also effective in hatching inhibition, achieving a 40% inhibition rate. This suggests that the affinity of compounds may vary at each stage, or perhaps the quantity of the active compound is not sufficient to achieve better effectiveness.

The compounds detected by GC-MS in the AQU fraction are listed in Table 4, and some of them have been recorded as nematocidal compounds. Hexadecanoic acid methyl ester (palmitic acid methyl ester), (9,12-Octadecadienoic acid (*Z*,*Z*)-methyl ester (linoleic acid methyl ester), and 2,4-Di-tert-butylphenol (2,4-DTBP) have been found to coincide in certain studies where their antagonist effectiveness against phyto-nematodes has been confirmed [39,40]. 2,4-DTBP is particularly noteworthy among them; it is a natural phenolic compound reported to have antifungal, insecticidal, and acaricidal properties. It occurs naturally in bacteria, plants, and fungi and has exhibited effectiveness against nematodes [41]. Li et al. [42] identified 2,4-DTBP in root exudates of tomato plants inoculated with *Bacillus cereus* and infected with *M. incognita*. In the same study, direct application of 2,4-DTBP in pot assays reduced the root galls (53.7%) and the presence of nematodes in tomato root tissue (67.5%) compared to controls. In another experiment, 2,4-DTBP and palmitic acid were among the major compounds of the *Moringa oleifera* ethyl acetate leaf extract, with high nematocidal effectiveness (100% at 10 mg/mL) against *N. aberrans* J_2_ [43]. Palmitic acid and linoleic acid were the predominant components found in a bioactive extract derived from cotton seed cake, which exhibited efficacy against *M. incognita* [39].

With respect to mushrooms, Akshaya et al. [40] tested the effectiveness of the EAC fraction obtained from *Ganoderma lucidum* culture cell-free against *M. incognita*. Their findings demonstrated hatching inhibition of 88.5% at 1000 ppm and a J_2_ mortality rate of 93.2%. These findings are comparable to those observed in our current study concerning J_2_, but they were not similar regarding eggs. Additionally, GC-MS analysis revealed the presence of 2,4-DTBP and palmitic acid methyl ester, also detected in our study. 2,4-DTBP has also been found as a chemical constituent of some oyster mushrooms [44,45]. Furthermore, there is evidence suggesting 2,4-DTBP’s role as an intermediary in lignin metabolism [46]. 

2,4-DTBP has been identified in the SMS of EMs. In a study by Fujita et al. [47], soil contaminated with *Alternaria brassicola* was treated with spent substrates of *H. marmoreous*, *L. edodes*, and *Cyclocybe cylindracea*. GC-MS analysis revealed the presence of 2,4-DTBP in the treated soil, while it was absent in the control. 2,4-DTBP has been reported in at least 169 different organism species, including bacteria, fungi, and plants [41]. Consequently, we cannot conclusively determine that the source of 2,4-DTBP in the AQU fraction is *P. ostreatus* alone, as the spent substrate is composed of plant material, mushroom residues, and microbial populations. 2,4-DTBP has also exhibited toxicity towards *C. elegans* FLN during soil fumigation trials [48]. Therefore, it is likely that the presence of 2,4-DTBP could potentially impact the survival of *P. redivivus*.

Oyster mushrooms have frequently been cited as a rich reservoir of fatty acids [49]. Linoleic acid is regarded as the primary nematocidal compound for trapping fungi [50]. Additionally, numerous studies have consistently demonstrated the NAT of fatty acids isolated from basidiomycetes against phyto-nematodes [20]. Palmitic and linoleic acids were detected in the current study; both compounds were found in root exudates from castor (*Ricinus communis* L.), which resulted in a reduction of *M. incognita* infection in an intercropping castor–tomato assay [51]. Another study suggests the NAT of linoleic acid and palmitic acid against *Heterodera zeae*, showing a 100% and 28% mortality rate at 0.5%, respectively [52]. According to Rocha et al. [53], palmitic acid demonstrates significant toxicity against various nematodes. It has been reported that palmitic acid is toxic to *C. elegans*, but its toxicity against *P. redivivus* is not well documented. Additional substances were identified within the AQU fraction, including 1,4:3,6-dianhydro-α-d-glucopyranose, 5-oxo-l-prolinate methyl ester, 1*H*-phenanthro[9,10-c] pyrazole, and 5-hydroxy-6,7,8-trimethoxy-2,3-dimethyl-chromone. However, there is currently no widely accepted evidence suggesting that these compounds exhibit NAT. According to Velasco-Azorsa et al. [54], there are only two reports of natural compounds with toxic potential against *N. aberrans*, i.e., capsidiol acts as a nematostatic agent, and various cadinenes cause mortality and inhibit egg hatching.

Nematode cuticle is probably the target; according to Caboni and Ntalli [55], cuticle is the first barrier that a nematocidal should break to enter the nematode body. Several groups of natural compounds have been reported as nematocidal, such as phenolic compounds, terpenes, alkaloids, saponins, and enzymes. The mode of action of nematocidal natural products is related to reactive oxygen species (ROS); for example, naphthoquinones on *Bursaphelenchus xylophilus* [56]. ROS can also lead to increased production of nitric oxide in cells, induce DNA damage, and excessive ROS triggers oxidative damage that restricts protein function and finally cell death. On the other hand, the terpenoid (+)-α-pinene was effective in inhibiting sites of acetylcholinesterase on *B. xylophilus*. An aldehyde compound (trans-2-hexenal) interfered with the normal metabolism and physiological processes of *B. xylophilus* [57]. Other mechanisms include the affectation of tyramine and octopamine receptors, DNA damage through the oxidative process, interference in the respiration chain, and dissolution of the cytoplasmic membrane, among others [58].

Wille et al. [59] assessed the potential of aqueous extracts from ten mushroom extracts on lettuce pots. *P. ostreatus* reached mortality and hatching inhibition at 100 and 98.4% of *M. incognita*, respectively. It is suitable to test fractions in situ experiments rather than a particular compound, due to the fact that some organisms, such as insects, could develop resistance slower to a mix of different substances than a single [60]. However, considering the abundance of studies reporting the nematocidal effectiveness of 2,4-DTBP and fatty acids, the assessment against *N. aberrans* in pots seems reasonable.

It is worth clarifying that the raw materials of the substrate can also have NAT naturally without the intervention of the fermentation process developed by the EMs. Castañeda-Ramírez et al. [35] evaluated the NAT of coffee pulp, pangola grass, and corn stover extracts, common materials in the cultivation of *Pleurotus* spp. The results revealed that the extracts had a mean effective concentration (EC_50_) of 1.19 (0.73–1.69) mg/mL on the mortality of *H. contortus* L_3_. Not only agro-industrial materials and mushrooms could provide metabolites, but the microbial community could also be responsible for releasing some compounds. According to Marques et al. [61], the microbial community in SMS could be composed of bacteria, archaea, and fungi. Ahlawat [62] identified fungi and bacteria in the spent substrate of two mushrooms. In the first, the presence of the fungi *Aspergillus fumigatus*, *Schizophyllum commune*, and *Pezizomycotina* sp. and the bacteria *Bacillus licheniformis*, *B. subtilis*, *Rummeliibacillus stabekisii*, and *Pseudomonas fluorescens* was observed in the SS of *P. sajor-caju*, while in the substrates of *V. volvacea*, *A. fumigatus*, *Paecilomyces variotii*, *Pichia guilliermondii*, and *B. pumilus* were found.

All extracts showed significant toxicity against *P. redivivus*, causing mortality rates exceeding 90% within 48 h at 20 mg/mL. Notably, extracts from non-spawned substrates exhibited the highest toxicity, with T2 demonstrating superior efficacy at both LC_50_ and LC_90_ values, respectively. In contrast, the spawned AT5 showed lower toxicity. This suggests that all substrates hold toxic compounds against *P. redivivus*. However, non-colonized substrates may either contain distinct compounds or higher concentrations of nematocidal active substances.

According to Soares et al. [15], nematocidal efficacy against *P. redivivus* was observed on the spent substrate of *Hypsizygus marmoreous*, and they attributed this effect to proteases. Armas-Tizapanti et al. [63] indicate that some *Pleurotus* species complement their food supply through the predation of FLNs and plant parasitic nematodes. Several studies suggest that fatty acids are the nematocidal principle of predation [13,64]. However, novel evidence revealed that 3-octanone is the nematocidal toxin of *P. ostreatus*; this compound is the major component in toxocysts, and upon contact with adults of *C. elegans*, it triggers rapid paralysis and disrupts cell membrane integrity. This disruption leads to an extracellular calcium influx into the cytosol and mitochondria, propagating cell death throughout the entire organism. Additionally, 3-octanone was able to paralyze *Rhabditis rainai*, *Oscheius mtriophila*, and *Pelodera teres*, the free-living nematodes [65]. This compound has also been detected in another *Pleurotus* species, and it has been associated with an aromatic and flavor volatile compound [66,67]. 3-octanone and 1-octen-3-ol were tested against J_2_ of *M. incognita*. While 3-octanone showed high effectiveness (4.6 µL), the other compound exhibited even better effectiveness (3.2 µL) [68].

Several natural compounds have negative effects against NTOs, such as furfural, which has an inhibitory effect on the growth of FLNs [57]. Our results are like those of other studies tested on NTOs. For example, the juice extract (12%) from *Agave tequilana* leaves by-product killed 100% of *P. redivivus* larvae [69]. Additionally, a hydrolate from *Artemisia absinthium*, previously reported for its nematocidal activity against *M. javanica*, demonstrated acute toxicity against *E. fetida* and *Allium cepa* [70]. For his part, Pino-Otín et al. [71] assessed the toxicity of a biopesticide derived from *Artemisia absinthium* on some NTOs; the results showed that the product is highly toxic against *Daphia magna* (LC_50_ = 0.236%). On the other hand, certain studies indicate the absence of damage to NTOs caused by natural products. A bioactive compound obtained from *Neonothopanus nambi* mushroom was effective against *M. incognita* but not toxic to the entomopathogenic nematode *Steinernema carpocapsae* [72]. According to Oka [73], FLNs are more tolerant to nematocides; furthermore, variations in sensitivity may be attributed to differences in affinity to the target enzyme, cuticle, body size, and other characteristics. The FLN *C. elegans* has also been tested as a model to assess several natural and synthetic nematocides. However, we proposed *P. redivivus* due to its shared clade 10 with some plant parasitic nematodes such as *Bursaphelenchus* spp. and *Aphelenchoides* spp. [74].

Finally, we obtained accurate and quick lectures to distinguish live and dead *N. aberrans* J_2_ by adding previously NaOH 0.1 N to the well. This technique has been used for determining the mortality of *H. glycines* [75], *M. incognita* [76], and *M. javanica* [77]. The successful application of this technique to *N. aberrans* was first achieved in the study conducted by Gómez-Gutiérrez et al. [78]. We hypothesize that the observed response to the stimulus may be attributed to the similarities between *Meloidogyne* spp. and *N. aberrans*. They have some similar characteristics, even though they share up to 40 protein domains and laterally acquired genes. Considering these similarities, it is promising to assess the nematocidal effectiveness of the AQU fraction against root knot nematodes (*Meloidogyne* spp.).

## 4. Materials and Methods

In vitro tests were performed at the Centro Nacional de Investigación Disciplinaria-Salud Animal e Inocuidad, Instituto Nacional de Investigaciones Forestales Agrícolas y Pecuarias (CENID-SAI, INIFAP), Morelos, México. The spent substrate was provided by El Colegio de la Frontera Sur (ECOSUR), Tapachula, Chiapas, México. Extracts and chemical fractions were obtained at the Phytochemical Department of the Centro de Investigación Biomédica del Sur (CIBIS) of the Instituto Mexicano del Seguro Social (IMSS), Morelos, Mexico.

### 4.1. Obtaining Hydroalcoholic Extracts of T5, T2, AT5, and AT2

In our study, the extracts were designated as T5, T2, AT5, and AT2, representing specific ratios of three different raw materials used to formulate the substrate. These raw materials were sourced from the local region in Tapachula, Chiapas, Mexico. T2 was formulated with coffee pulp (67.67%), pangola grass (16.66%), and 16.66% maize cob waste, whereas T5 was prepared with an equal distribution for each material (33.3%). Notably, extracts bearing the “A” prefix were exclusively derived from substrates that had been spawned with *P. ostreatus* mycelium. Previously, SMS was ground in a PULVEX mill (Mexico city, México); then, 150 g of SMS were mixed with 1.2 L of methanol-water (60:40). After 24 h, the mixture was filtrated with filter paper whatman #4 and concentrated in a rotary evaporator Heidolph (Schwabach, Germany) at 45 °C, 120 rpm, and reduced pressure conditions. Finally, the remanent was lyophilized in a LABCONCO (Kansas City, MO, USA) freeze dryer [14].

### 4.2. Liquid–Liquid Partition of AT5 Extract

Hexane, dichloromethane, and ethyl acetate were utilized as solvents in a liquid–liquid extraction procedure. Initially, AT5 was dissolved in water (500 mL), followed by the addition of hexane (500 mL), and the mixture was allowed to stand for 15 min. Subsequently, the separated phases were recovered, and dichloromethane was added to the aqueous phase to obtain the dichloromethane (DM) fraction. Next, ethyl acetate was added to the aqueous phase to yield the acetate fraction (EAC) and the aqueous fraction (AQU). The hexane fraction yielded a low yield, and thus it was excluded from further consideration. The obtained volumes were concentrated and ultimately subjected to lyophilization, following the procedure outlined by Nakamura et al. [79]. This sequential process resulted in the following three distinct fractions: the aqueous (AQU) phase, the dichloromethane (DM) phase, and another from ethyl acetate (EAC).

### 4.3. Nematocidal Test on Nacobbus Aberrans J_2_

#### 4.3.1. Obtaining Eggs and J_2_ of *N. aberrans*

**Figure 4:**
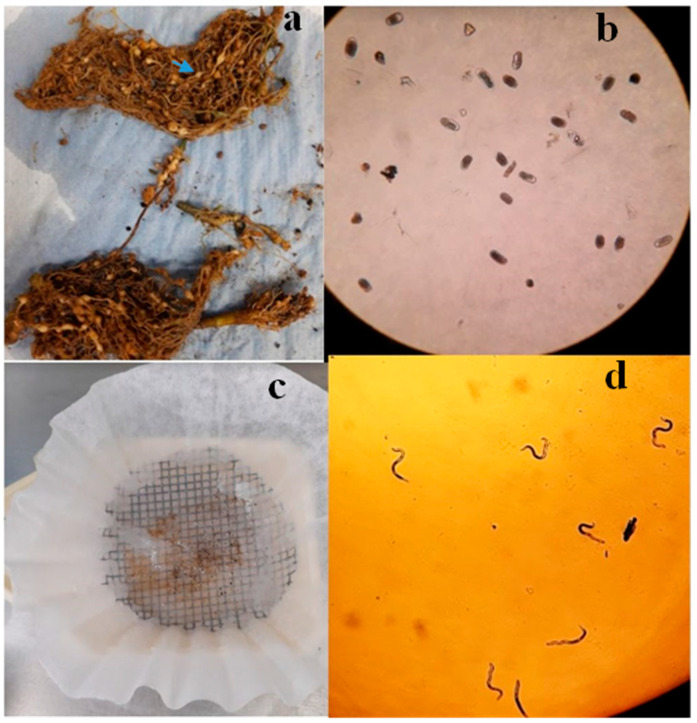
(**a**) Fresh roots infected with *N. aberrans*; blue arrow shows a knot induced by an adult female (40× magnification); (**b**) Fresh eggs extracted from the roots; (**c**) Fresh eggs incubated in distilled water; (**d**) J_2_ hatched.

Eggs from *N. aberrans* were obtained from the gall-infested roots of tomato plants (*Solanum lycopersicum*), cultivated at the Instituto Politécnico Nacional, CIIDIR-IPN, Michoacán. The roots were initially washed with tap water and then cut into fragments measuring 1–2 cm. These fragments were subsequently immersed in a 1.5% NaClO solution, shaken for 3 min, and the resulting mixture was filtered through 75, 45, and 37 μm sieves before being rinsed with distilled water. The suspension retained on the sieve was then subjected to centrifugation at 3000 rpm. The precipitated phase was treated with 5 mL of MgSO_4_ (1.18 specific gravity), and the solution was centrifuged immediately at 3000 rpm for 3 min. The upper phase was collected on a 37 µm sieve, and the retained eggs were recovered after undergoing 4–5 washes with distilled water [80]. Freshly collected eggs were subsequently incubated in distilled water for 8 days at a temperature of 25 ± 1.0 °C to obtain J_2_ (Figure 4), as described by Vázquez-Sánchez et al. [81].

#### 4.3.2. Larvicidal Activity of Extracts against J_2_ of *N. aberrans*

To assess the nematocidal effect of extracts against *N. aberrans* (J_2_), a random experimental design was employed for this experiment. The hydroalcoholic extracts were dissolved in sterile distilled water; the latter was used as a negative control. The in vitro test was carried out on 96-well microtiter plates. Each well contained a mixture of 50 µL of the corresponding treatment, 30 µL of chloramphenicol (at a concentration of 333.33 µg/mL), and 20 µL of distilled water containing 100 J_2_. The final concentrations were set at 20–0.625 mg/mL. The plates were sealed and incubated for 72 h in a humid chamber at room temperature. Subsequently, before examining each well, 10 µL of 1N NaOH was added. Later, a microscope Carl Zeiss Primo Star (Jena, Germany) at a 40× magnification was employed to observe the twisted appearance of live larvae in response to the chemical stimulus, as described by Gómez-Gutiérrez et al. [78]. The results were expressed in terms of LC_50_ and LC_90_. Four replicates were conducted for each treatment, and the experiment was repeated three times.

### 4.4. Nematocidal Activity of AQU, DM, and EAC Fractions against J_2_ of N. aberrans

AQU, DM, and EAC fractions were assessed for their nematocidal effectiveness against J_2_, aiming to screen their potential efficacy. Previously, fractions were diluted with 8% methanol (MET) at a final concentration of 2 mg/mL. The MET at 4% served as a negative reference and confrontation, and the criteria for determining J_2_ mortality were the same as described in the test for extracts. Three replicates were performed, and the response variable was percent mortality.

### 4.5. Comparing the Effectiveness of AQU Fraction and Commercial Nematicide against N. aberrans

The effectiveness of the AQU fraction was compared with a commercial nematicide (Nematrol Plus^®^ Michoacán, Mexico). The AQU fraction was diluted with 8% methanol to reach a final concentration of 2 mg/mL. Similarly, chitosan (CHI) at 6 mg/mL, as tested by Gómez-Gutiérrez et al. [78], and MET at 4% were utilized as references for both eggs and J_2_. The confrontation process and criteria for determining J_2_ mortality were the same as described in the bioassay of hydroalcoholic extracts. Percent mortality was the response variable. Each treatment underwent six replicates for eggs and 15 replicates for J_2_.

For the hatching experiment, 20 µL of egg suspension (250 eggs), 30 µL of chloramphenicol, and 50 µL of the fraction were deposited in a well. Each treatment had six replicates. The eggs were then incubated at room temperature, and after 8 days, the number of eggs and J_2_ were observed under a Carl Zeiss Primo Star microscope (Jena, Germany) at a 40X magnification. The hatching inhibition percentage was calculated using Equation (1) [12].

(1) $$Hatching\;inhibition\left(\%\right)=100-\left[\left[\frac{{NJ}_{2}}{\left({NJ}_{2}+NE\right)}\right]\times 100\right]$$

where NJ_2_ = number of hatching J_2_ and NH = number of eggs.

### 4.6. Gas Chromatography–Mass Spectrometry Analysis of AQU Fraction

The AQU fraction was subjected to GC-MS analysis through an external service provided by the Centro de Investigación y de Estudios Avanzados del Instituto Politécnico Nacional in Merida, Mexico. The objective of this analysis was to identify the compounds present within the fraction. Previously, 2 mg of dry AQU fraction was dissolved in 1 mL of methanol. The analysis was conducted using a Thermo Scientific TRACE GC equipped with an ITQ900 ion trap mass detector (Thermo Electron Corporation, Milan, Italy). Helium was used as the carrier gas at a flow rate of 1 mL/min. The column employed was a TRACE-5MS (30 m length, 0.25 μm film thickness, and 0.25 mm internal diameter). The GC was outfitted with a split-less injector maintained at 270 °C.

The oven program was set as follows: initially held at 50 °C for 1 min, followed by a temperature ramp in three steps: from 50 to 300 °C at a rate of 7 °C/min, and finally held at 300 °C for 5 min, resulting in a total chromatographic time of 56 min. Mass spectra were acquired via electron impact ionization at 70 eV, and the detector (Thermo Electron Corporation, Milan, Italy) was configured in TIC/Scan (Total Ion Current) mode, scanning from 50 to 650 m/z at a rate of 0.2 scans per second (Dwell). The transfer line and ion source temperatures were maintained at 270 °C and 200 °C, respectively. The raw data obtained from the analysis were processed using Xcalibur^TM^ software version 4.0 (Thermo Fisher Scientific, Waltham, MA, USA.

### 4.7. Nematocidal Test on Panagrellus redivivus Mix Population

#### 4.7.1. *Panagrellus redivivus* Culturing

Nematodes were cultured in oat-water medium; subsequently, ten grams of the medium were taken and washed with M9 buffer over 75, 45, and 37 μm sieves [32]. The retained solution was centrifuged (3500 rpm/3 min), and the sediment was re-suspended in MgSO_4_ (1.18 specific gravity). Subsequently, it was centrifuged again, and the upper phase was washed with M9 buffer over the 37 µm sieve to remove excess MgSO_4_; then, the larvae retained on the sieve were recovered and conserved in M9 buffer. From the obtained nematode suspension, a count was conducted. For this, ten aliquots of 10 μL volume were taken and deposited onto a slide in triplicate. A larvae count was performed on each slide, and an average was obtained, which was then scaled up to the total volume of the suspension. Following this, a mixed population of nematode suspension containing one larva/μL was prepared.

#### 4.7.2. Nematocidal Activity of Extracts against *P. redivivus* Larvae

Concentrations of extracts from 20–0.625 mg/mL were tested, with four replicates per treatment and three repetitions of the experiment, and M9 buffer was used as solvent. In vitro tests were carried out in 96-well microtiter plates, each well being considered an experimental unit. The treatments were randomly assigned to the wells, and 50 µL of nematode suspension and 50 µL of corresponding treatment were mixed in each well. The response variable was the percentage of mortality after 48 h, determined by observing the motility and the response to physical stimuli. The LC_50_ and LC_90_ were estimated.

### 4.8. Statistical Analysis

All experiments were conducted using a completely randomized design. Data collection was performed using the KDSmart application v3.4.0 (DArT, 2021). Subsequently, mortality data from extracts confronted against *N. aberrans* and *P. redivivus* were subjected to a probit analysis using the drc package from the R environment. The Shapiro–Wilk test was performed to verify normality of the recorded mortality rates for J_2_ exposed to fractions, as well as the data from the test of the AQU fraction compared against chitosan, were subjected to a one-way ANOVA. Statistical comparisons of means were carried out using the Tukey test with a significance level of α = 0.05 [82].

## 5. Conclusions

One of the most widely cultivated genera is *Pleurotus* spp., and several of its species have been associated with nematocidal properties. In the present study, we confirmed the NAT of hydroalcoholic extracts from *P. ostreatus* spent substrate. At the in vitro test, the AQU fraction was equivalent in J_2_ mortality to the commercial nematicide (chitosan), while in egg hatching inhibition reached 40%. The results from this study suggest that in the three fractions, there are antagonist compounds against *N. aberrans* J_2_. This means that low-cost solvents are effective in obtaining nematocidal compounds. Considering *P. redivivus* as a beneficial and bioindicator organism, we conclude that non-fermented substrates are more toxic than colonized, so SMS is a suitable alternative because it provides food and nematocidal compounds. With the ban on several active ingredients, it is urgent to explore alternatives to control phyto-parasitic nematodes. Additional studies can be proposed on another FLN, like *C. elegans*, to verify if extracts are toxic to other nematodes. Finally, there are several factors found in the agricultural field that are often not adequately reproduced experimentally, and hence, in vitro tests should be validated. The effectiveness reached with the AQU fraction allows us to propose the assessment (J_2_ mortality, suppression of infection, egg laying, and egg hatching inhibition) and elicitor roles of 2,4-DTBP and fatty acids through the experimental design of mixtures.

## Figures and Tables

**Table 1:** Lethal mean concentration (mg/mL) of extracts tested against J_2_ of *N. aberrans*.

Treatments	LC_50_ (CI 95%)	LC_90_ (CI 95%)	*X* ^2^	*p*-Value
AT2	4.11 (3.89–4.32)	49.8 (42.85–56.73)	6.90	0.141
AT5	2.73 (2.64–2.82)	9.83 (9.26–10.4)	8.05	0.089
T2	1.80 (1.75–1.86)	6.12 (5.80–6.44)	6.54	0.162
T5	0.96 (0.91- 1.01)	5.56 (5.16–5.97)	6.40	0.172

CI = Confidence interval; *X*^2^ = Chi square.

**Table 2:** Mean mortality of *N. aberrans* J_2_ exposed in vitro to AT5 fractions.

Treatments	Mean Mortality (%) ± Sd
AQU	87.95 ^a^ ± 5.26
EAC	62.43 ^b^ ± 10.22
DM	46.55 ^b^ ± 7.33
MET 4%	1.88 ^c^ ± 1.62

DM: Dichloromethane fraction; EAC: Ethyl acetate fraction; AQU: Aqueous fraction; MET: Methanol control (4%); Means with different letters show statistical differences according to the Tukey test (α = 0.05), F-statistic: 83.11 on 3 and 8 DF, *p* < 0.001.

**Table 3:** Compounds identified in the AQU fraction by GC-MS analysis. Identification was conducted by comparing the electron ionization (EI) mass spectrum available in the NIST mass spectral database and the Wiley library.

RT (min)	Compound	MW (gr/mol)	Structure
7.51	1,4:3,6-dianhydro-α-d-glucopyranose	144.12	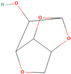
9.81	5-oxo-l-prolinate methyl ester	143.14	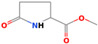
11.44	2,4-Di-tert-butylphenol	206.32	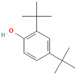
12.97	1*H*-phenanthro[9,10-c] pyrazole	218.0	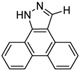
15.92	hexadecanoic acid methyl ester	270.45	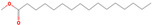
17.52	9,12-octadecadienoic acid (*Z*,*Z*) methyl ester	294.47	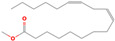
19.63	5-hydroxy-6,7,8-trimethoxy-2,3-dimethyl-chromone	280.27	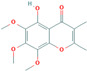
23.36	17α(*H*), 21β(*H*)-25,30-bisnorhopane	384.7	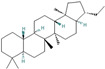

**Table 4:** Lethal mean concentration (mg/mL) of extracts tested against *P. redivivus* larvae.

Treatments	LC_50_ (CI 95%)	LC_90_ (CI 95%)	*X* ^2^	*p*-Value
AT2	4.83 (4.28–5.38)	25.12 (19.34–30.92)	8.88	0.064
AT5	8.56 (7.04–10.09)	93.15 (49.06–137.24)	4.30	0.366
T2	1.37 (1.25–1.51)	3.99 (3.43–4.56)	4.04	0.40
T5	1.82 (1.64–2.00)	5.34 (4.56–6.13)	3.32	0.506

CI = Confidence interval; *X*^2^ = Chi square.

## Data Availability

The original contributions presented in the study are included in the article/Supplementary Material; further inquiries can be directed to the corresponding authors.

## Supplementary Materials

The following supporting information can be downloaded at: https://www.mdpi.com/article/10.3390/plants13131777/s1. Figure S1: LC_50_ of chitosan against J_2_ of *N. aberrans*. Range concentration [0.187–6 mg/mL], 72 h post-confrontation.
