# Supplementary material for: Hydroalcoholic Extracts from Pleurotus ostreatus Spent Substrate with Nematocidal Activity against Nacobbus aberrans Phytonematode and the Non-Target Species Panagrellus redivivus
Source: Plants (Basel). 2024 Jun 27;13(13):1777. doi: 10.3390/plants13131777 (PMC11244132; doi:10.3390/plants13131777)
Supplement: Supplementary file 1 [file plants-13-01777-s001.zip › plants-3031878-supplementary.pdf]

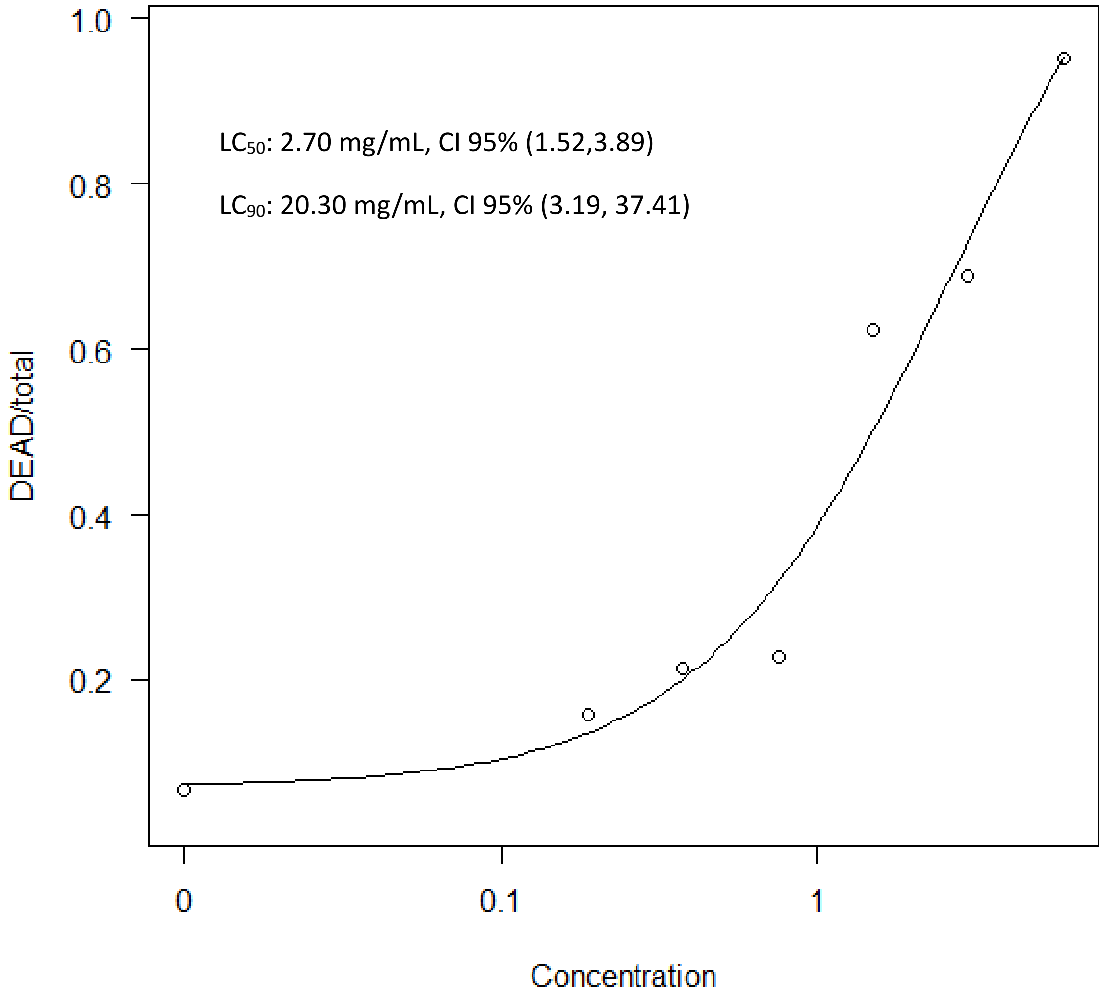

Figure S1: LC_{50} of chitosan against J_2 of *N. aberrans*. Range concentration [0.187-6 mg/mL], 72 h post-confrontation.
